# Hyaluronidase Reverses the Effect of Hyaluronic Acid Without Destroying Normal Skin Structure

**DOI:** 10.1111/jocd.70870

**Published:** 2026-05-14

**Authors:** Bin Yin, Zhiling Jiang, Jian Shi, Hui Wang, Peng Wang, Chiyu Jia

**Affiliations:** ^1^ The First Affiliated Hospital, Center of Burn & Plastic and Wound Healing Surgery, Hengyang Medical School University of South China Hengyang Hunan China; ^2^ School of Medicine Xiamen University Xiamen China

**Keywords:** collagen synthesis, fibroblast, hyaluronic acid, hyaluronidase

## Abstract

**Background:**

Dermal fillings based on hyaluronic acid have been widely used in facial rejuvenation. A number of studies have reported the beneficial effect of hyaluronidase in saving the complications of hyaluronic acid. However, the effects of hyaluronidase on dermal matrix, skin structure, and fibroblast function are unknown.

**Method:**

In this study, hyaluronic acid and hyaluronidase were injected into rat skin, skin samples were collected at multiple time points, and the changes of skin structure, fibroblast function, and extracellular matrix gene and protein expression were detected.

**Result:**

The results in vivo showed that hyaluronidase can effectively degrade exogenously injected hyaluronic acid in rats without causing significant changes in dermal matrix and skin structure. The results in vitro showed that hyaluronidase had no significant effect on proliferation, apoptosis, and collagen synthesis of fibroblasts.

**Conclusion:**

This study provided a theoretical basis for the clinical application of hyaluronic acid and hyaluronidase. In vivo and in vitro experiments confirmed that hyaluronidase can degrade exogenous hyaluronic acid without damaging the skin structure, dermal matrix, and fibroblast function.

## 1 Introduction

Dermal fillings based on hyaluronic acid have been widely used in facial rejuvenation because of their low incidence of complications, low price, and can be reversed by hyaluronidase [1]. However, with the increase in the use of hyaluronic acid fillers, its complications are also frequently reported [2]. Some studies have reported that hyaluronic acid‐based fillers can stimulate procollagen and various growth factors in aging skin and increase the proliferation of dermal fibroblasts, vascular system and epidermis [3].

In addition, typical complications of hyaluronic acid caused by injection techniques include superficial placement, uneven placement, granuloma reaction, and skin necrosis. Most of the complications will disappear over time. However, severe cosmetic deformities require more aggressive treatment with adjuvant drugs such as hyaluronidase [4].

Hyaluronidase is a naturally occurring enzyme that can degrade hyaluronic acid and is often used to correct filling complications caused by hyaluronic acid [4]. A number of studies have reported the beneficial effect of hyaluronidase in saving hyaluronic acid complications. However, it is unknown whether it can effectively reverse the extracellular matrix stimulation of hyaluronic acid, whether it will affect the structure of normal skin, and whether it will affect the function of fibroblasts.

In this study, we injected hyaluronic acid and hyaluronidase into rat skin, collected skin biopsy samples at multiple time points, and analyzed the changes in skin structure, fibroblast function, and extracellular matrix gene and protein expression. The purpose of this study was to evaluate the reversal effect of hyaluronidase on hyaluronic acid and its effect on normal skin.

## 2 Materials and Methods

This study was divided into two parts: the first part was the in vivo study, which aimed to explore the reversal effect of hyaluronidase on hyaluronic acid and its effect on normal skin. The second part referred to the in vitro study, which aimed to explore the effect of hyaluronidase on the function of human dermal fibroblasts.

### 2.1 In Vivo Study

#### 2.1.1 Study Design

**FIGURE 1:**
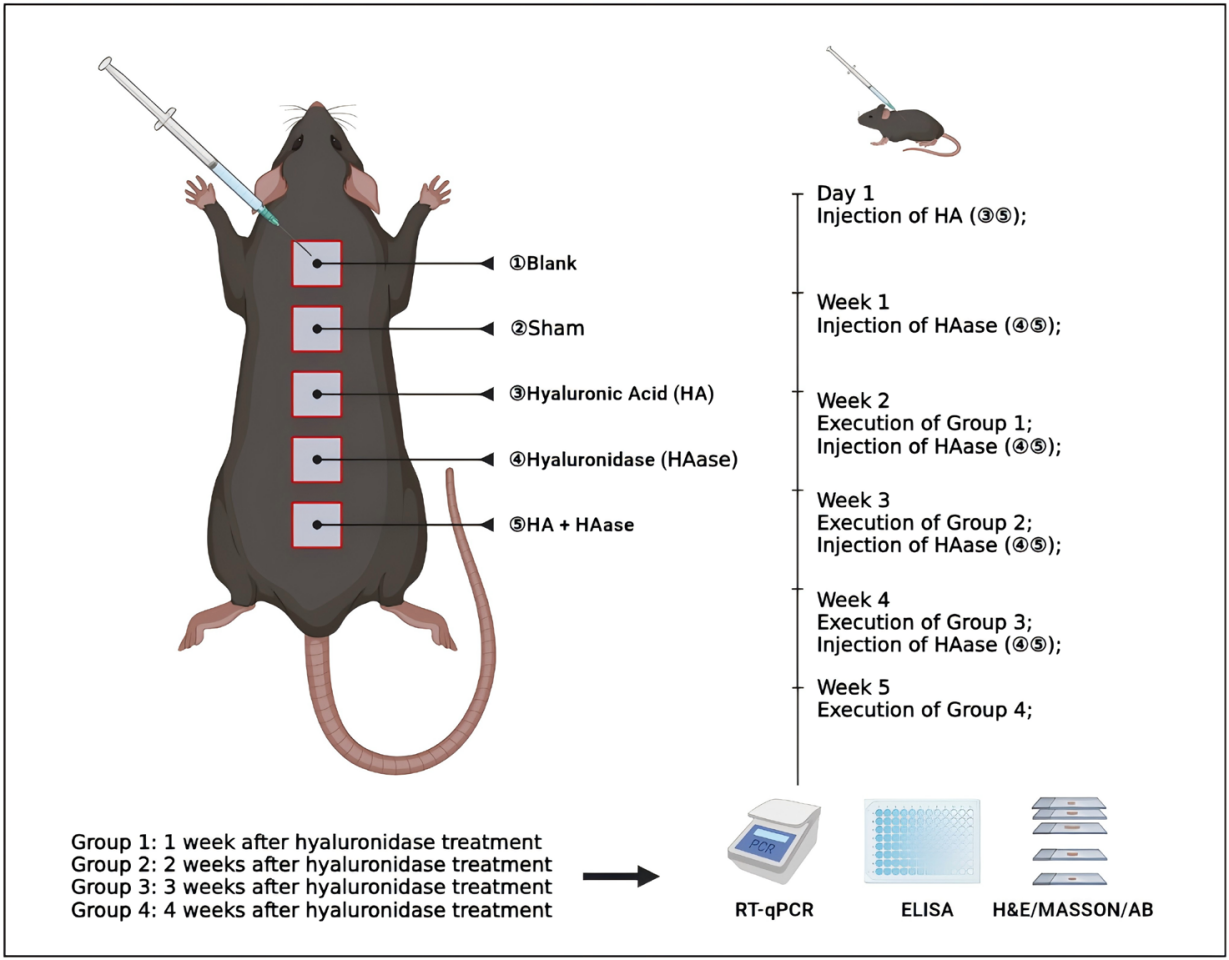
The experimental design of in vivo study.

This study was approved by the Animal Ethics Committee of Xiamen University. Sprague–Dawley rats (*n* = 6 per group) aged 2–4 months were raised in the Experimental Animal Center of Xiamen University. After anesthesia, the back of these rats was treated with the following: 50‐μL intradermal injection of saline (sham group), HA (High molecular weight (≥ 1000 kDa), non‐crosslinked, Bloomage Freda Biopharm Co. Ltd., China), HAase (150 IU/mL, Shanghai No. 1 Biochemical & Pharmaceutical Co. Ltd., China), HA + HAase, or no treatment (blank group). Intradermal injections were performed using a 30‐gauge needle at a depth of approximately 1–2 mm. The detailed experimental design is shown in Figure 1.

#### 2.1.2 Hematoxylin–Eosin Staining (H&E), Masson and Alcian Blue Staining

The animals were sacrificed for the first, second, third, and fourth weeks after hyaluronidase injection. The skin samples were collected and stored in 4% polyformaldehyde. Then H&E, MASSON, and Alcian blue staining were performed. The degradation of hyaluronic acid and the change of skin structure were observed under a microscope.

#### 2.1.3 Enzyme‐Linked Immunosorbent Assay (Elisa)

Skin samples were collected and then transported in liquid nitrogen. The skin tissue was ground up and then centrifuged with 5000 g for 5 min at 4°C to collect the supernatant. According to the manufacturer's instructions, the Rat COL1 (Collagen Type I) ELISA Kit and Rat COL3 (Collagen Type III) ELISA Kit (Elabscience, China) were used to detect the collagen content of the supernatant.

### 2.2 In Vitro Study

#### 2.2.1 Study Design

**FIGURE 2:**
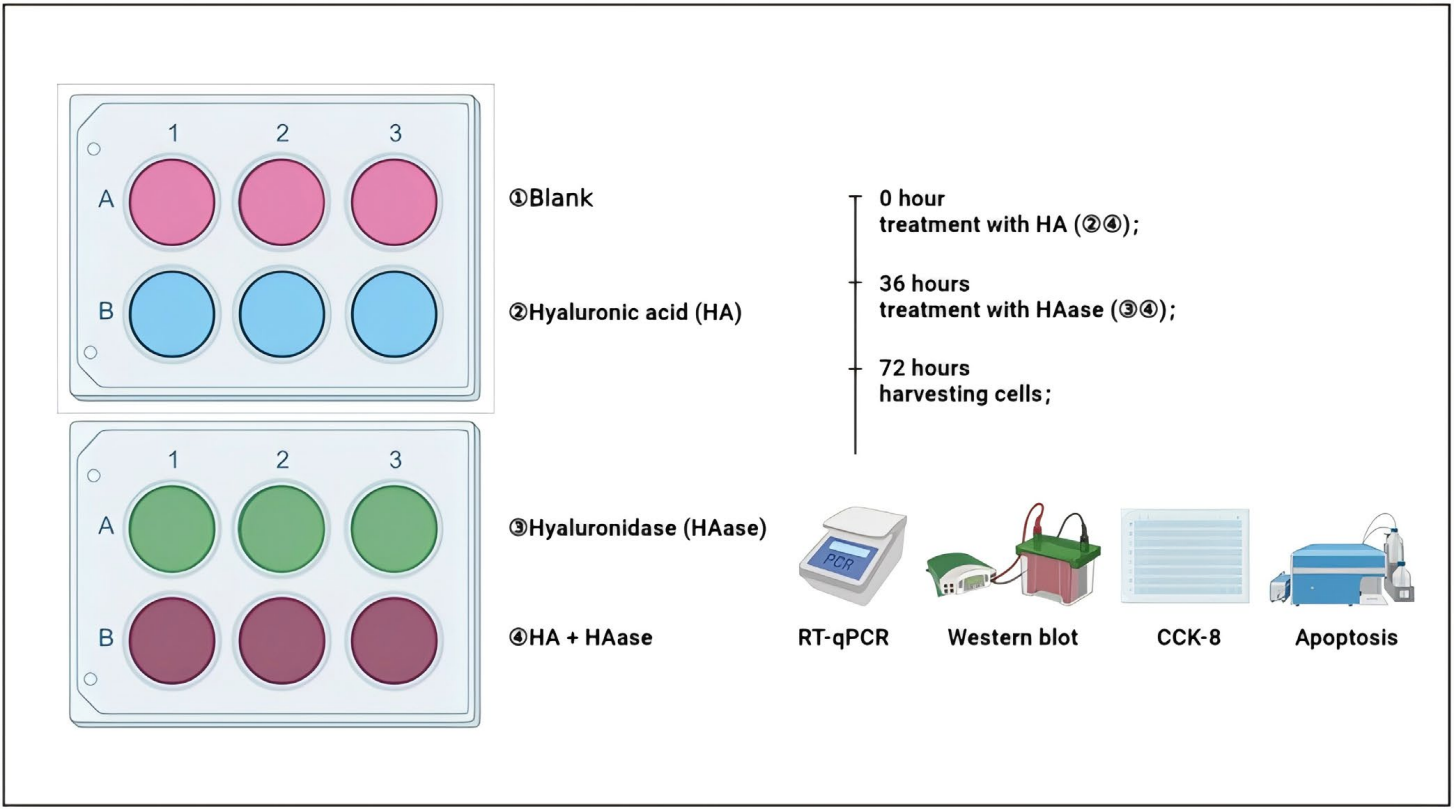
The experimental design of in vitro study.

Each recruited patient obtained informed consent, and this study was approved by the Ethics Committee of Xiang'an Hospital affiliated with Xiamen University. Normal skin specimens were collected from patients who underwent surgery in Xiang'an Hospital affiliated with Xiamen University. A previous study has described in detail the isolation of primary fibroblasts [5]. The fibroblasts were treated with the following: 50‐μL HA (as above), HAase (as above), HA + HAase, or no treatment (blank group). The detailed experimental design is shown in Figure 2.

#### 2.2.2 Ethics Statement

The animal study was reviewed and approved by the Animal Ethics Committee of Xiamen University. All procedures involving animals were conducted in accordance with institutional guidelines for the care and use of laboratory animals. The human study was reviewed and approved by the Ethics Committee of Xiang'an Hospital affiliated with Xiamen University. The study was conducted in accordance with the principles of the Declaration of Helsinki.

#### 2.2.3 Informed Consent

Informed consent was obtained from all individual participants from whom normal skin specimens were collected.

### 2.3 Cell Proliferation

The proliferation of fibroblasts was detected using CCK8 kit (LABLEAD, China). According to the manufacturer's instructions, 3 × 10^3^ fibroblasts were inoculated into each well of the 96‐well plates and pre‐cultured to about 40% confluence. CCK‐8 reagent was added to each well at different time points, and then the 96‐well plate was incubated in a dark place for 2 h. Finally, the absorbance at 450 nm was detected by Microplate reader, and the proliferation curve was drawn.

### 2.4 Cell Apoptosis

Annexin V‐FITC/PI apoptosis detection kit (YEASEN, China) was used to detect the apoptosis of fibroblasts. According to the manufacturer's instructions, the fibroblasts were collected and re‐suspended in 100 μL binding buffer and incubated with 5 μL Annexin V‐FITC and 10 μL PI for 10–15 min in the dark. Finally, flow cytometry was used to detect the apoptosis of the cells, and the apoptosis rate was calculated by Flowjo software.

### 2.5 RNA Extraction and RT‐qPCR

According to the manufacturer's instructions, Cell/Tissue Total RNA Kit (YEASEN, China) was used to extract the total RNA. qPCR SYBR Green Master Mix (YEASEN, China) and real‐time PCR system (CFX96 Touch, Bio‐Rad, USA) were used to perform PCR. The sequences of primers are as follows: COL1A1(Mouse) Forward Primer‐GCTCCTCTTAGGGGCCACT, Reverse Primer‐CCACGTCTCACCATTGGGG; COL3A1(Mouse) Forward Primer‐CTGTAACATGGAAACTGGGGAAA, Reverse Primer‐CCATAGCTGAACTGAAAACCACC; COL1A1(Human) Forward Primer‐GAGGGCCAAGACGAAGACATC, Reverse Primer‐CAGATCACGTCATCGCACAAC; COL3A1(Human) Forward Primer‐GGAGCTGGCTACTTCTCGC, Reverse Primer‐GGGAACATCCTCCTTCAACAG. And GAPDH was used as an internal control. The relative expression of genes was determined by the standard 2^−ΔΔCt^ method.

### 2.6 Statistical Analysis

The statistical comparisons between the groups were performed by Student's *t*‐test or one‐way analysis of variance (ANOVA) using GraphPad Prism V. 9 (GraphPad Software, USA). *p* < 0.05 indicated a significant difference.

## 3 Results

### 3.1 The Reversal Effect of Hyaluronidase on Hyaluronic Acid

**FIGURE 3:**
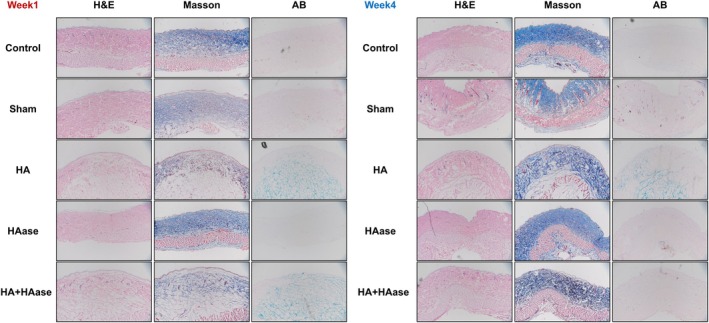
H&E, MASSON, and AB staining of rats skin after HA or HAase injection.

To understand the reversal effect of hyaluronidase on exogenously injected hyaluronic acid in rat skin, the skin tissue injected with hyaluronidase was stained. In the first week, there was a large amount of hyaluronic acid deposition in the dermis of both HA and HA + HAase groups confirming the presence of the injected material. At the fourth week, most of the hyaluronic acid was retained in the HA group, while the exogenous hyaluronic acid was completely dissolved and the skin restored the normal structure in the HA + HAase group, which was not significantly different from that in the control group (Figure 3).

### 3.2 The Effect of Hyaluronidase on Normal Skin of Rat

**FIGURE 4:**
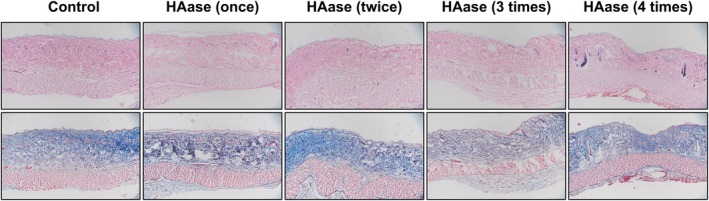
H&E and MASSON staining of rats' skin after HAase injection.

To explore the effect of hyaluronidase on the normal skin structure of rats, we recorded the changes of skin structure after hyaluronidase injection. The results of H&E and Masson staining showed that slight indentation could be observed in some skin tissues after the third and fourth injection. No other significant changes in skin structure were found in each group. There was no obvious inflammatory cell infiltration in each group (Figure 4).

### 3.3 The Effect of Hyaluronidase on Extracellular Matrix of Rat Skin

**FIGURE 5:**
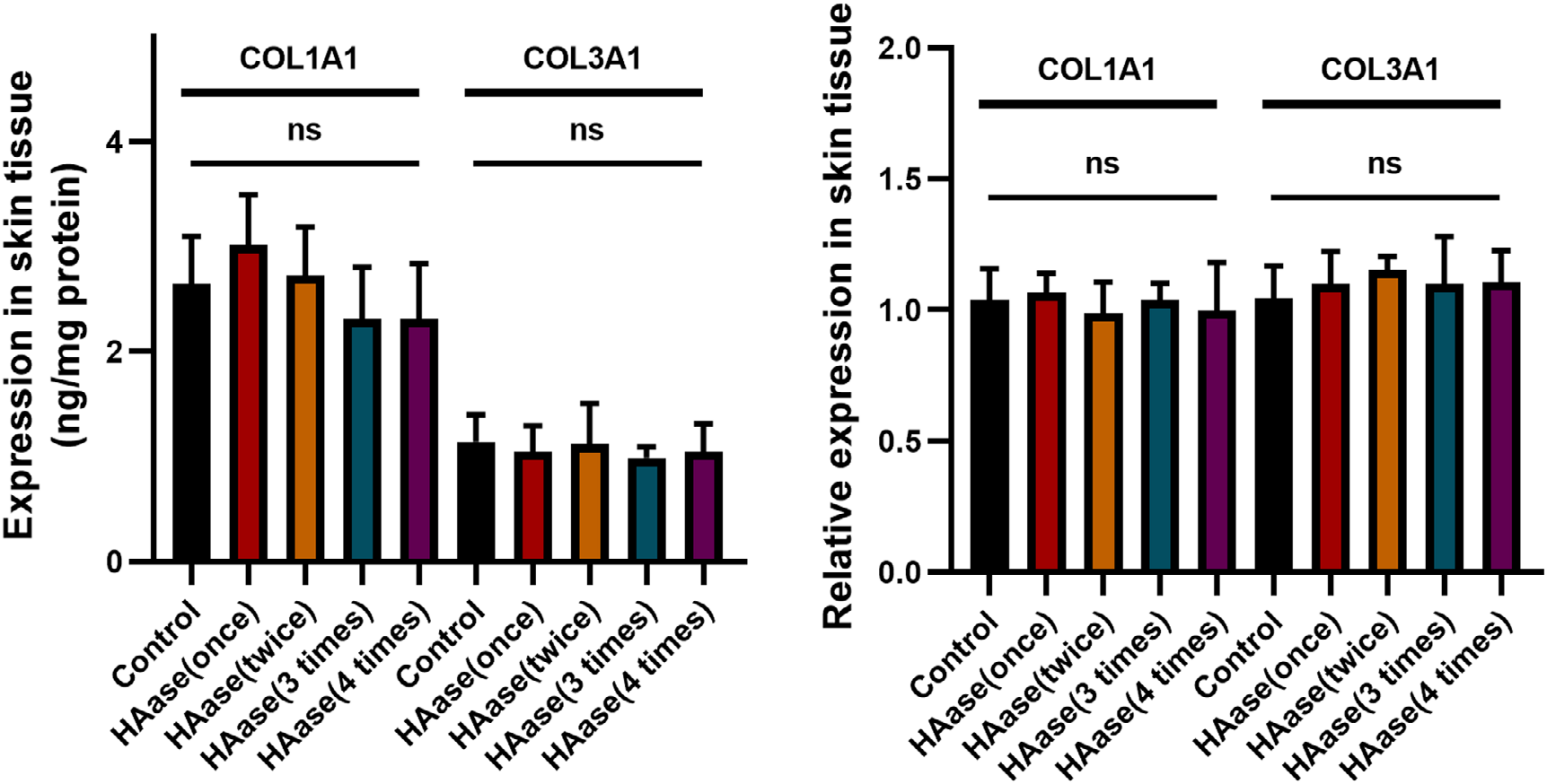
The expression of collagens of rats' skin after HAase injection.

To further understand the effect of hyaluronidase on the extracellular matrix of rat skin, the changes of collagen I and III after injection of hyaluronidase were detected. The results of ELISA and PCR showed that there was no significant change in collagen expression after multiple injections of hyaluronidase compared to the control group, indicating no detrimental effect on endogenous ECM components (Figure 5).

### 3.4 The Effect of Hyaluronidase on Proliferation and Apoptosis of Human Dermal Fibroblasts

**FIGURE 6:**
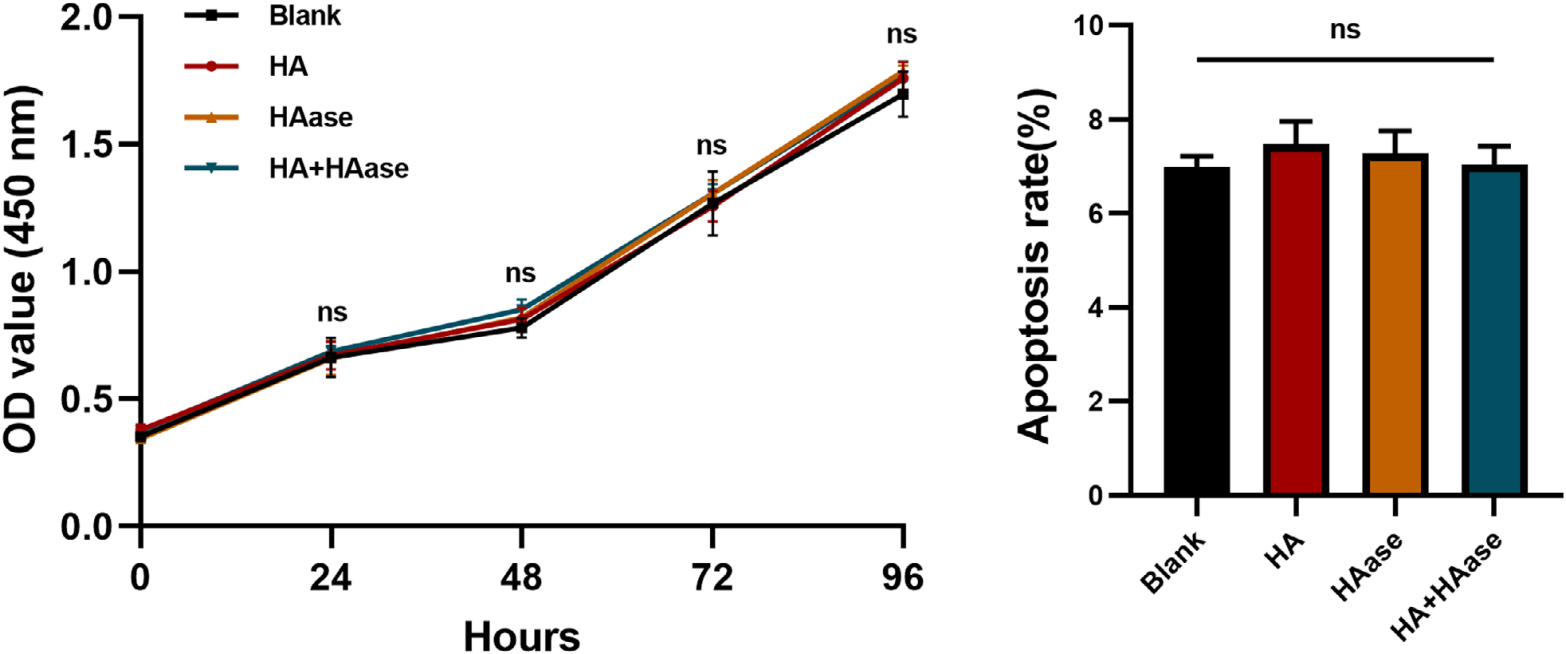
The proliferation and apoptosis of human dermal fibroblasts after HA and HAase treatment.

Fibroblasts are the main cells in the dermis. We further explored the effect of hyaluronidase on proliferation and apoptosis of fibroblasts. The results showed that neither hyaluronic acid nor hyaluronidase affected the proliferation and apoptosis of fibroblasts compared to the blank control group (Figure 6).

### 3.5 The Effect of Hyaluronidase on Collagen Synthesis of Human Dermal Fibroblasts

**FIGURE 7:**
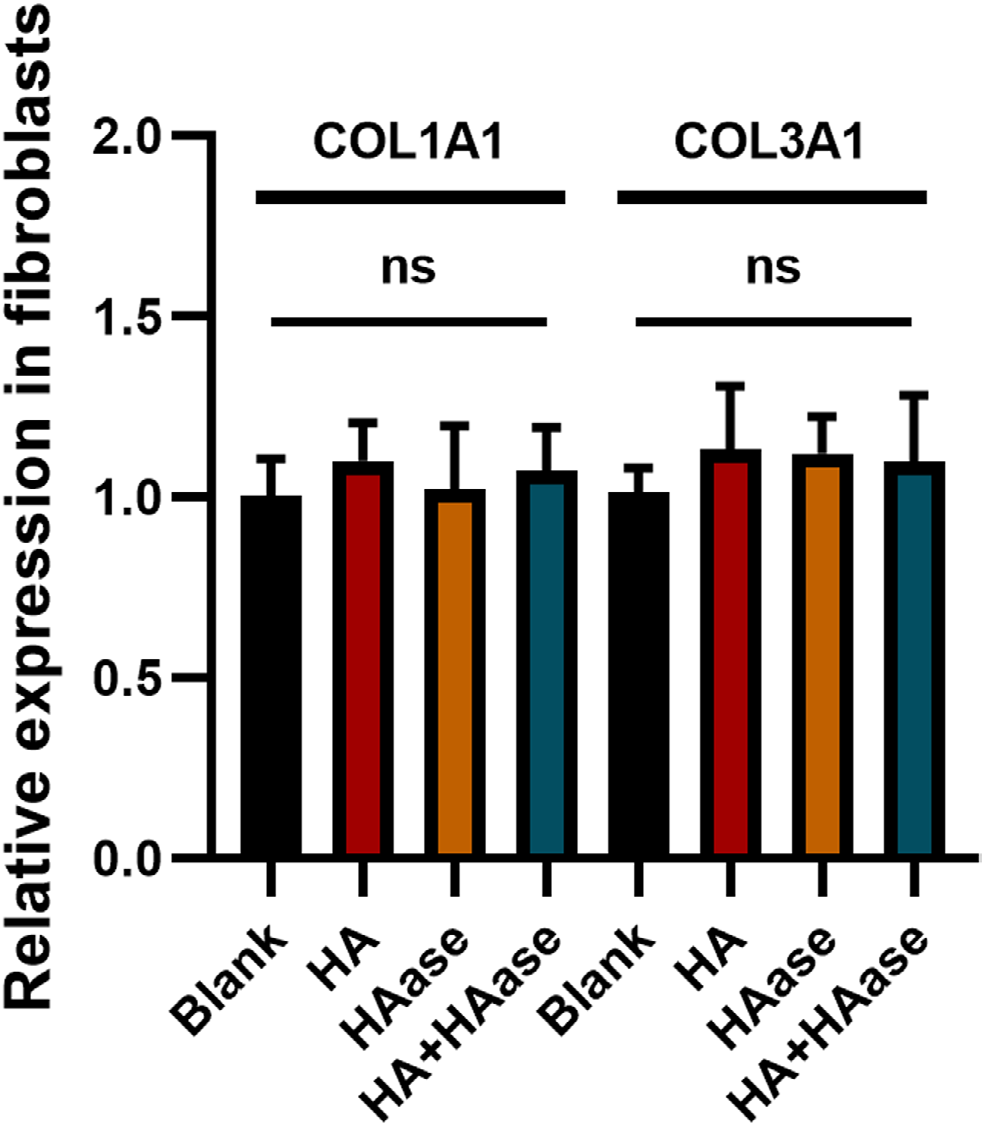
The collagen synthesis of human dermal fibroblasts after HA and HAase treatment.

Collagen synthesis is the main function of dermal fibroblasts. RT‐qPCR showed that hyaluronidase did not affect the collagen synthesis of fibroblasts compared to the control groups (Figure 7).

## 4 Discussion

In recent years, minimally invasive injection techniques for correcting skin aging have become increasingly accepted [6]. Hyaluronic acid fillers are characterized by low immunogenicity, ease of use, and gradual degradation by the body. However, complications arising from hyaluronic acid fillers have also increased [7, 8, 9]. Hyaluronidase, as an antagonist of hyaluronic acid, is frequently used to address these complications [10]. It remains unclear whether repeated hyaluronidase injections have negative effects on normal skin structure and function [11]. In this study, we explored the effects of hyaluronic acid and hyaluronidase on normal skin both in vivo and in vitro. Our key finding is that hyaluronidase effectively degraded exogenously injected HA without significantly damaging the normal skin structure, endogenous ECM components, or fibroblast function.

Our in vivo results demonstrated that co‐injection or subsequent injection of HAase successfully reversed the persistence of exogenous HA filler by the fourth week, restoring the skin architecture to a state comparable to the control. This aligns with the primary clinical use of HAase [4, 10]. Importantly, injections of HAase alone did not cause significant alterations in skin structure (beyond occasional slight indentations potentially due to volume or multiple injections) or in the expression of key endogenous ECM components like Collagen I and III. This suggests that under the conditions used, the enzymatic activity of HAase is predominantly directed towards the exogenous HA substrate rather than the native tissue architecture. The slower degradation kinetics observed in our rat model compared to typical clinical resolution might be attributed to species differences in hyaluronidase activity, tissue metabolism, the specific physicochemical properties of the HA filler used, and the injection volume relative to tissue size.

The potential for HAase to affect cellular functions was further investigated in vitro using human dermal fibroblasts. Our data clearly showed that exposure to HA, HAase, or their combination did not significantly alter fibroblast proliferation, apoptosis, or collagen synthesis capacity. This indicates a lack of direct cytotoxicity or functional modulation of these key skin cells by the enzyme at the concentrations tested. This finding is crucial for clinical safety, as it suggests that HAase application is unlikely to impair the biological functions of dermal fibroblasts responsible for tissue maintenance and repair.

Our results regarding the lack of significant impact of HAase on endogenous collagen are consistent with some previous studies. Gerber et al. demonstrated time‐ and dose‐dependent effects of hyaluronidase on various HA fillers in vitro, focusing on filler degradation itself rather than native tissue components [12]. Similarly, Buhren et al. established a standardized in vitro model for assessing HA filler degradation by hyaluronidase, again primarily addressing the filler material [13]. Our study extends these findings by specifically examining the tissue response in vivo and cellular response in vitro, reinforcing the notion that HAase's action can be confined to the exogenous filler. However, the optimal dosing and potential long‐term effects of repeated HAase applications, especially in compromised tissue, require further investigation, as highlighted by Hong et al. [14].

The limitations of this study include the following points: First, the rat model used, while common, has different skin characteristics compared to humans, which may affect the clinical relevance of the results. Second, the sample size was relatively small, and the experimental conditions may not fully replicate actual clinical scenarios, impacting the reliability and translatability of the findings. Additionally, the observation period was short, only 4 weeks, which does not allow for the assessment of the long‐term effects and chronic side effects of hyaluronidase. Although human skin samples were used in the in vitro studies, primary human scar fibroblasts were not utilized, which may affect the generalizability of the results. Furthermore, while no significant allergic reactions were observed, potential impacts on the immune system may require further investigation. Moreover, changes in the molecular weight of hyaluronic acid were not assessed in the experiment, which could influence the understanding of hyaluronidase's effects on hyaluronic acid metabolism. Lastly, although fibroblast proliferation and apoptosis were evaluated, their migration and repair capabilities were not comprehensively assessed, which is crucial for understanding the overall impact of hyaluronidase. Importantly, a key limitation of this study is the use of non–cross‐linked HA rather than cross‐linked HA fillers, which are more commonly used in clinical practice. Cross‐linked HA exhibits distinct rheological properties and degradation kinetics, which may influence its interaction with hyaluronidase and subsequent tissue responses. Therefore, caution should be exercised when extrapolating these findings to clinical settings. Future studies incorporating clinically relevant cross‐linked HA fillers are warranted to further validate and extend the present findings. These limitations suggest that future research should address these issues to enhance the clinical relevance and applicability of the study findings.

## 5 Conclusion

In conclusion, our study provides experimental evidence that hyaluronidase can effectively reverse the effects of exogenously injected hyaluronic acid filler in a rat model without causing significant damage to the normal skin structure, endogenous extracellular matrix, or the function of human dermal fibroblasts in vitro. These findings support the clinical use of hyaluronidase as a safe reversal agent for HA fillers. Future studies should focus on validating these results in models closer to human clinical settings and exploring the effects in different tissue conditions.

## Author Contributions

Bin Yin and Zhiling Jiang put forward the conception and design of the study. Bin Yin and Jian Shi completed the relevant experiments. Peng Wang and Hui Wang collected the specimens and analyzed the data. Bin Yin and Zhiling Jiang explained the data. Bin Yin and Jian Shi drafted the article. Chiyu Jia and Bin Yin revised the article. All authors had reviewed the final manuscript and approved it for submission.

## Funding

Health Research Project of Hunan Provincial Health Commission, China (Grant 20257724). Project supported by the Natural Science Foundation of Hunan Province, China (Grant 2026JJ70062).

## Conflicts of Interest

The authors declare no conflicts of interest.

## Data Availability

The data that support the findings of this study are available from the corresponding author upon reasonable request.
